# CD226 Deletion Reduces Type 1 Diabetes in the NOD Mouse by Impairing Thymocyte Development and Peripheral T Cell Activation

**DOI:** 10.3389/fimmu.2020.02180

**Published:** 2020-09-04

**Authors:** Melanie R. Shapiro, Wen-I Yeh, Joshua R. Longfield, John Gallagher, Caridad M. Infante, Sarah Wellford, Amanda L. Posgai, Mark A. Atkinson, Martha Campbell-Thompson, Scott M. Lieberman, David V. Serreze, Aron M. Geurts, Yi-Guang Chen, Todd M. Brusko

**Affiliations:** ^1^Department of Pathology, Immunology and Laboratory Medicine, University of Florida Diabetes Institute, Gainesville, FL, United States; ^2^Department of Pediatrics, University of Florida, Gainesville, FL, United States; ^3^Stead Family Department of Pediatrics, University of Iowa Carver College of Medicine, Iowa City, IA, United States; ^4^The Jackson Laboratory, Bar Harbor, ME, United States; ^5^Department of Physiology, Medical College of Wisconsin, Milwaukee, WI, United States; ^6^Department of Pediatrics, Medical College of Wisconsin, Milwaukee, WI, United States

**Keywords:** type 1 diabetes, Sjögren’s disease, costimulatory receptors, CD8^+^ T cells, CD226

## Abstract

The costimulatory molecule CD226 is highly expressed on effector/memory T cells and natural killer cells. Costimulatory signals received by T cells can impact both central and peripheral tolerance mechanisms. Genetic polymorphisms in *CD226* have been associated with susceptibility to type 1 diabetes and other autoimmune diseases. We hypothesized that genetic deletion of *Cd226* in the non-obese diabetic (NOD) mouse would impact type 1 diabetes incidence by altering T cell activation. CD226 knockout (KO) NOD mice displayed decreased disease incidence and insulitis in comparison to wild-type (WT) controls. Although female CD226 KO mice had similar levels of sialoadenitis as WT controls, male CD226 KO mice showed protection from dacryoadenitis. Moreover, CD226 KO T cells were less capable of adoptively transferring disease compared to WT NOD T cells. Of note, CD226 KO mice demonstrated increased CD8^+^ single positive (SP) thymocytes, leading to increased numbers of CD8^+^ T cells in the spleen. Decreased percentages of memory CD8^+^CD44^+^CD62L^–^ T cells were observed in the pancreatic lymph nodes of CD226 KO mice. Intriguingly, CD8^+^ T cells in CD226 KO mice showed decreased islet-specific glucose-6-phosphatase catalytic subunit-related protein (IGRP)-tetramer and CD5 staining, suggesting reduced T cell receptor affinity for this immunodominant antigen. These data support an important role for CD226 in type 1 diabetes development by modulating thymic T cell selection as well as impacting peripheral memory/effector CD8^+^ T cell activation and function.

## Introduction

MHC class I hyper-expression and autoreactive CD8^+^ T cell infiltration in pancreatic islets constitute two prominent histological features of type 1 diabetes and are thought to be involved in the direct cytotoxic killing of β-cells (1, 2). Other immune cell subsets, including CD4^+^ T cells, B cells, monocytes, and natural killer (NK) cells have also been reported to contribute to insulitis and β-cell destruction (3, 4). Genetic studies have identified 57 independent genetic loci beyond MHC class II that contribute to the development of type 1 diabetes (5). These variants are thought to contribute to defects in immune tolerance, with a number of additional loci also associated with β-cell fragility and susceptibility to immune surveillance and attack (5–13). While many of the candidate single nucleotide polymorphisms (SNPs) and causative variants are being identified with greater resolution (14), there remain a number of outstanding questions regarding how candidate loci impact gene expression and function within tissues and cell types, as well as over various developmental time points and activation states during disease pathogenesis.

A SNP in *CD226* (rs763361) has been associated with genetic susceptibility to multiple autoimmune diseases including type 1 diabetes, multiple sclerosis, and rheumatoid arthritis (15). The SNP results in a missense mutation leading to a glycine to serine substitution at position 307 and is located proximally to two intracellular phosphorylation sites (Tyr322 and Ser329) of CD226 (16, 17). Hence, it has previously been shown that the rs763361 risk allele increases phosphorylation status of downstream signaling mediators, such as Erk, augmenting CD226 activity in human CD4^+^ T cells (18). Notably, the *Idd21.1* risk locus of the non-obese diabetic (NOD) mouse model of type 1 diabetes contains the *Cd226* gene and is orthologous to the 18q22.2 region containing the human *CD226* gene (19), thereby making the NOD mouse a superb *in vivo* model of CD226 activity in the context of autoimmunity.

CD226 functions as an activating costimulatory receptor in the immunoglobulin superfamily (20) that is expressed largely on effector and memory T cells and NK cells (21, 22). CD226 activity is antagonized by an inhibitory counterpart, T cell Immunoreceptor with Ig and ITIM domains (TIGIT), which functions as a negative regulator with expression enriched on regulatory T cells (Tregs) (22) and NK cells (23). CD226 and TIGIT function in an analogous manner to the more widely studied CD28:CTLA-4 costimulatory axis (24), to promote activation or inhibition via immunoreceptor tyrosine-based activation (ITAM) or inhibitory motifs (ITIM), respectively. CD226 activation is reported to be dependent on homodimerization and binding to cognate ligands, including CD155 (PVR) and CD112, on antigen-presenting cells (APCs) (23, 25, 26). CD226 has been demonstrated by fluorescence resonance energy transfer to be inhibited in *cis* through interactions with TIGIT (27).

Costimulatory molecules are known to influence central tolerance by fine-tuning T cell receptor (TCR)-mediated signaling that defines thresholds for thymocyte selection (28). CD226, in particular, has been implicated in supporting the survival of CD4^+^CD8^+^ double positive (DP) as well as CD4^+^ single positive (SP) thymocytes (29). The interaction between CD226 and CD155 has also been shown to drive the thymic retention and negative selection of CD8^+^ SP thymocytes, shaping the CD8^+^ T cell repertoire (30, 31). Together, these studies suggest that the balance of CD226:TIGIT signaling may influence positive and negative selection of thymocytes; however, the impact of this signaling pathway on the autoreactive T cell repertoire remains poorly defined.

Similar to other costimulatory molecules, CD226 and TIGIT are also known to regulate peripheral tolerance by impacting T cell and NK cell activation and function. CD226 promotes, while TIGIT inhibits, CD4^+^ T cell proliferation and differentiation into a Th1 phenotype (32), as well as CD8^+^ T cell (20, 27) and NK cell cytotoxicity (33, 34). While the roles of CD226 and TIGIT in type 1 diabetes pathogenesis remain unclear, blockade of CD226 has been shown to protect from experimental autoimmune encephalitis (EAE), another autoimmune mouse model in which disease pathogenesis is thought to be primarily T cell-mediated (35).

Therefore, we sought to understand how CD226 and TIGIT impact central and peripheral tolerance mechanisms in the context of type 1 diabetes. We hypothesized the genetic deletion of *Cd226* would attenuate disease development, whereas disruption of *Tigit* would promote type 1 diabetes. Herein, we present the impact of genetic disruption of these costimulatory receptors on the incidence of type 1 diabetes in the NOD mouse model. We further demonstrate mechanisms by which the elimination of CD226 impacts both central and peripheral T cell activation.

## Materials and Methods

### Mice

CRISPR-Cas9 technology was used to knockout (KO) *Cd226* and *Tigit*, as previously described (36), on the NOD/ShiLtJ (NOD, IMSR Cat# JAX:001976, RRID:IMSR_JAX:001976) background. The following guide sequences were used to target genes of interest: *Cd226*, AAGTCCTGAGTCAGCGGCCA; *Tigit*, CTGCTGCTTCCAGTCGACTT. Founders were backcrossed to NOD/ShiLtJ mice for two generations prior to intercrossing heterozygous (HET) mice, to prevent potential off-target deletions from inheritance in the experimental breeders. Animals were bred and housed in specific pathogen-free facilities at the University of Florida, with food and water available *ad libitum*. All studies were conducted in accordance with protocols approved by the University of Florida Institutional Animal Care and Use Committee (UF IACUC) and in accordance with the National Institutes of Health Guide for Care and Use of Animals.

### Genotyping

Offspring from CD226 or TIGIT HET x HET mating pairs were genotyped using custom Taqman SNP assays according to manufacturer’s protocol (Thermo Fisher Scientific). Reporter 1 was marked with VIC and reporter 2 with FAM dyes to distinguish alleles and quantitative PCR performed on a Roche LightCycler 480.

*Cd226* Forward primer sequence: GGGAGCATGAAGAGC ATCCT, *Cd226* Reverse primer sequence: GCGACATCTGTAA AGTCCTGAGT, *Cd226* Reporter 1 sequence (wild-type, WT): CAAATGCCATGGCCGCT, *Cd226* Reporter 2 sequence (KO): CAAATGCCATGCCGCT.

*Tigit* Forward primer sequence: ATCTTACAGTGTCACTT CTCCTCTGA, *Tigit* Reverse primer sequence: TCAACACTA TAAATGGCCAGAAGCT, *Tigit* Reporter 1 sequence (WT): AAGTGACCCAAGTCGACTG, *Tigit* Reporter 2 sequence (KO): AAGTGACCCATCGACTG.

### Measurement of Diabetes Incidence

Littermate CD226 or TIGIT WT, HET, and KO animals were monitored weekly starting at 9 weeks of age for onset of diabetes. Blood glucose levels were measured with AlphaTrak2 glucometer, and animals with blood glucose over 250 mg/dL were re-checked the following day. Those with a second consecutive reading over 250 mg/dL were considered diabetic.

### Histology, Insulitis, Sialoadenitis, and Dacryoadenitis Scoring

Pancreata, submandibular salivary glands, and extraorbital lacrimal glands were removed at necropsy and fixed in 10% neutral buffered formalin overnight. Samples were embedded in paraffin and a total of three sections (4 μm) at 250 μm steps of each pancreas, or one section (5 μm) of paired salivary and lacrimal glands, were stained with hematoxylin and eosin. For pancreata, whole digital slide scans were performed using an Aperio CS scanner (Leica Biosystems) and for salivary and lacrimal glands, images were obtained with a PathScan Enabler IV slide scanner (Meyer Instrument). Insulitis scoring was performed by a blinded observer. At least 45 islets per mouse were scored using previously published criteria (37). An islet was defined as at least 10 endocrine cells. Briefly, a score of 0 = no infiltration, 1 = peri-insulitis, 2 ≤50% of islet infiltrated, 3 ≥50% of islet infiltrated with immune cells. Salivary and lacrimal infiltration was quantified using standard focus scoring as previously described (38). Foci were defined as ≥50 mononuclear cells and total number of foci were normalized by surface area of the section.

### Adoptive Transfer

Pooled spleen and non-draining lymph nodes of age-matched female pre-diabetic (8–12 weeks of age) CD226 WT and CD226 KO mice were processed to single cell suspensions, and CD4^+^ and CD8^+^ T cells were separated by magnetic bead isolation (Miltenyi Biotec). 2 × 10^6^ CD4^+^ T cells and 1 × 10^6^ CD8^+^ T cells suspended in 100 μL PBS were transferred by tail vein injection into 6–8 week-old female NOD.SCID recipients, and diabetes incidence was monitored weekly for up to 20 weeks post-transfer.

### Flow Cytometry

Spleen, thymus, and lymph nodes were processed using frosted glass slides and passed through a 40 micron filter, for lymphocyte analysis, or a 70 micron filter, for APC analysis, to create a single cell suspension. Red blood cells were lysed with ammonium-chloride-potassium buffer prior to staining for flow cytometry analysis. Pancreas was minced and digested with 0.5 mg/mL Collagenase IV (Life Technologies) for 35 minutes at 37°C prior to passing through a 70 micron filter. Ficoll density gradient centrifugation was performed to enrich for immune infiltrates within the pancreatic digest. Samples were stained with Fixable Live/Dead Near IR (Invitrogen), and Fc receptors were blocked with anti-CD16/32 (Clone 2.4G2, BD Biosciences Cat# 553142, RRID:AB_394657). Antibodies used included CD3e-BV605 (145-2C11, BioLegend Cat# 100351, RRID:AB_2565842), CD4-PerCP/Cy5.5 (RM4-5, Thermo Fisher Scientific Cat# 45-0042-82, RRID:AB_1107001) or –Pacific Blue (RM4-4, BioLegend Cat# 116007, RRID:AB_11147758), CD8a-eFluor450 (53-6.7, Thermo Fisher Scientific Cat# 48-0081-82, RRID:AB_1272198) or –BV711 (53-6.7, BioLegend Cat# 100759, RRID:AB_2563510), NKp46-FITC (29A1.4, BioLegend Cat# 137606, RRID:AB_2298210), CD44-PE (IM7, BioLegend Cat# 103008, RRID:AB_312959), CD62L-APC (MEL-14, BioLegend Cat# 104412, RRID:AB_313099), CD226-AF647 (10E5, BioLegend Cat# 128808, RRID:AB_1227541), TIGIT-PerCP/eFluor710 (GIGD7, Thermo Fisher Scientific Cat# 46-9501-82, RRID:AB_11150967), CD96-PE (3.3, BioLegend Cat# 131705, RRID:AB_1279389), MHC Class II (I-Ad)-FITC (AMS-32.1, BD Biosciences Cat# 553547, RRID:AB_394914) or -APC (AMS-32.1, Thermo Fisher Scientific Cat# 17-5323-80, RRID:AB_10717083), CD11c-eFluor450 (N418, Thermo Fisher Scientific Cat# 48-0114-82, RRID:AB_1548654), CD155-PE (4.24.1, BioLegend Cat# 132205, RRID:AB_1279105), CD112-FITC (502-57, LifeSpan Cat# LS-C210245, RRID:AB_2857846), and CD5-AF488 (53-7.3, BioLegend Cat# 100612, RRID:AB_493165). Pancreas and pancreatic lymph node (pLN) cells were stained with MHC I (H-2Kd) IGRP (206-214) tetramer obtained from NIH Tetramer Facility for 30 minutes at 4°C. The same batch of tetramer was used throughout the study in order to avoid variability in fluorescence intensity due to batch-to-batch differences. Data were acquired on an LSRFortessa (BD Biosciences) and analyzed with FlowJo (v10.3, Tree Star, RRID:SCR_008520).

### Statistical Analysis

Data were analyzed using GraphPad Prism software version 7.0 (RRID:SCR_002798). Data are presented as mean ± standard deviation (SD) unless otherwise specified. Survival curves of CD226 or TIGIT WT, HET, and KO littermates were compared using log-rank (Mantel–Cox) test. Insulitis scoring was compared between WT and KO mice via chi-square test. Sialoadenitis, dacryoadenitis, and flow cytometric data of WT and KO mice were compared via Mann–Whitney test. Flow cytometric data from WT, HET, and KO mice were analyzed using Kruskal–Wallis with Dunn’s multiple comparisons test. Comparisons of CD226, CD112, and CD155 expression between cell subsets within the same mouse were performed using Friedman test with Dunn’s multiple comparisons test. *p* < 0.05 was considered significant.

## Results

### CD226 KO Protected NOD Mice From Type 1 Diabetes

**FIGURE 1:**
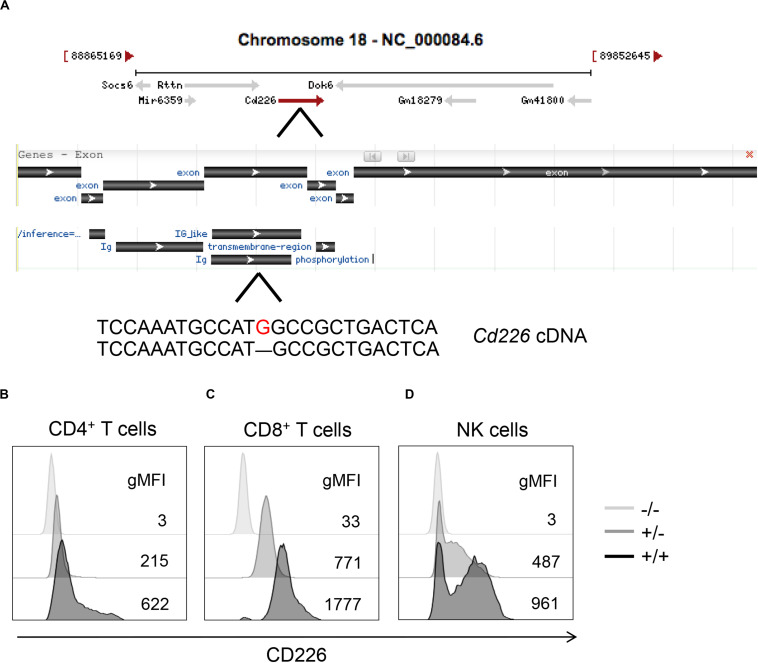
CRISPR/Cas9 targeting methods for generation of CD226 KO strain. **(A)** A guide RNA was used to target the fourth exon of *Cd226*, inducing a single base pair deletion (red) that led to a frameshift and premature termination of CD226 protein translation. Modified image from Gene NCBI. Histograms showing CD226 expression in splenic **(B)** CD4^+^ T cells, **(C)** CD8^+^ T cells, and **(D)** NK cells of 12-week-old female, pre-diabetic WT (black), HET (gray), and KO (light gray) mice.

A single base pair deletion in the immunoglobulin-like region of *Cd226* was induced via CRISPR/Cas9 targeting (Figure 1A), creating a frameshift which resulted in premature translation termination and lack of membrane CD226 protein expression in KO mice, as confirmed by flow cytometry of CD4^+^ T cells, CD8^+^ T cells and NK cells (Figures 1B–D). A two-base pair deletion in the immunoglobulin-like region of *Tigit* was induced (Supplementary Figure S1A), resulting in a loss of membrane TIGIT protein expression, which was most apparent in NK cells (Supplementary Figures S1B–D). Animals HET for CD226 or TIGIT showed an intermediate level of protein expression, suggestive of a gene dosage effect (Figures 1B–D and Supplementary Figures S1B–D).

**FIGURE 2:**
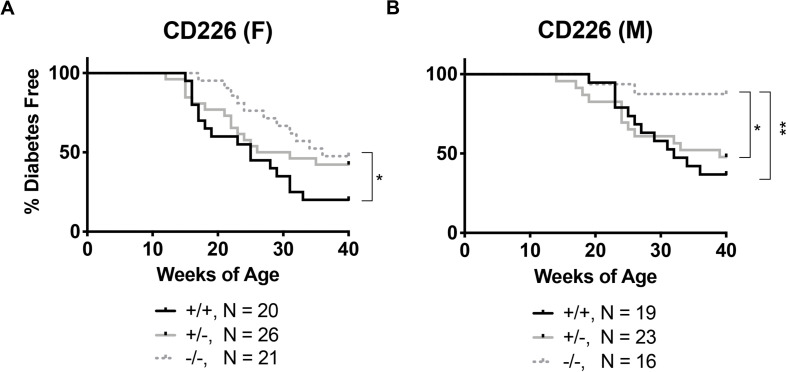
CD226 KO NOD mice are protected from type 1 diabetes. Disease incidence was monitored weekly, with diabetes defined as two consecutive daily blood glucose readings >250 mg/dL. CD226 KO (dotted lines) significantly lowers disease incidence in **(A)** females and **(B)** males as compared to WT (solid black) or HET (solid gray) mice. Log-rank (Mantel–Cox) test; *p < 0.05; **p < 0.01. Female: +/+, *n* = 20; +/-, *n* = 26; –/–, *n* = 21; Male: +/+, *n* = 19; +/-, *n* = 23; –/–, *n* = 16.

To measure disease incidence, littermate CD226 and TIGIT WT, HET and KO blood glucose levels were monitored weekly from 9 to 40 weeks of age. In our colony, diabetes develops in approximately 80% of female (Figure 2A) and 60% of male (Figure 2B) WT NOD mice. Importantly, we observed that CD226 KO showed significantly reduced diabetes incidence, with 52% of KO females (Figure 2A, *N* = 11/21, *p* = 0.021) and 13% of KO males (Figure 2B, *N* = 2/16, *p* = 0.004) developing disease. Female and male CD226 HET mice showed similar kinetics of early disease development as compared to WT mice, suggesting that a single copy of the gene was sufficient to promote type 1 diabetes (Figure 2). TIGIT HET and KO females and males showed similar diabetes incidence to WT littermates (Supplementary Figure S2). These data suggest a role for CD226 in promoting type 1 diabetes development, and thus, we chose to focus on this strain for the remainder of our studies.

### Pancreatic and Lacrimal Inflammation Are Reduced in CD226 KO NOD

**FIGURE 3:**
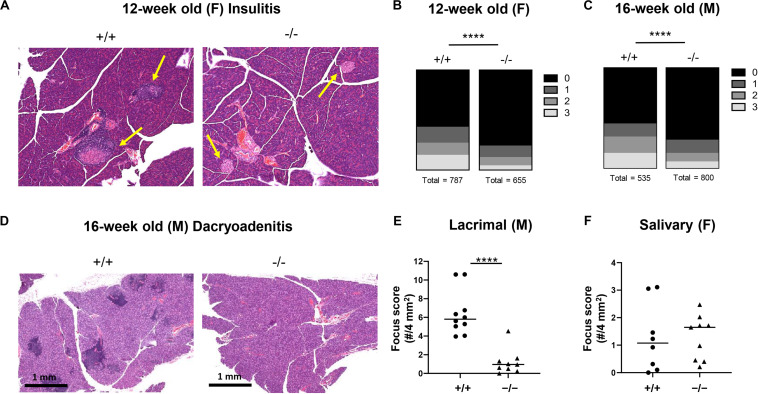
CD226 KO mice have reduced insulitis and dacryoadenitis. At least 45 islets per mouse were analyzed from three sections 250 microns apart. **(A)** Representative images of hematoxylin and eosin-stained pancreata from 12-week old female CD226 WT and KO mice. Yellow arrows denote islets. **(B)** Total number of islets categorized by insulitis score for pre-diabetic 12-week old females or **(C)** pre-diabetic 16-week old males. Chi-square test; ****p < 0.0001. Female: *N* = +/+, 10; –/–, 10. Male: *N* = +/+, 7; *N* = –/–, 10. **(D)** Representative images of hematoxylin and eosin-stained lacrimal glands showing numerous inflammatory foci in CD226 WT, but not KO lacrimal glands. Bars are 1 mm. **(E)** Dacryoadenitis scoring from 10–16 week old male CD226 WT and KO mice. **(F)** Sialoadenitis scoring in 10–14 week old female CD226 WT and KO mice. Symbols represent individual mice. Lines are medians. Mann–Whitney test; ****p < 0.0001.

NOD mice spontaneously develop inflammation of the pancreatic islets as well as the salivary and lacrimal glands, leading to the manifestation of both type 1 diabetes and Sjögren’s associated pathologies (39). Salivary and lacrimal infiltration are primarily observed in female and male NOD mice, respectively (40). We observed significantly reduced infiltration of the islets in pre-diabetic 12-week old female (Figures 3A,B) and 16-week old male (Figure 3C) CD226 KO versus WT NOD mice, likely close to potential disease onset (Figures 2A,B). Lacrimal inflammation was significantly reduced in male CD226 KO as compared to WT mice (Figures 3D,E), though the extent of salivary gland inflammation was consistent between age-matched female CD226 WT and KO mice (Figure 3F). These findings suggest that CD226 may influence the pathogenesis of both type 1 diabetes and Sjögren’s disease and provided impetus for understanding the immunological mechanisms of protection against autoimmunity in the CD226 KO NOD model.

### T Cell Expression of CD226 Contributes to Type 1 Diabetes Pathogenesis

**FIGURE 4:**
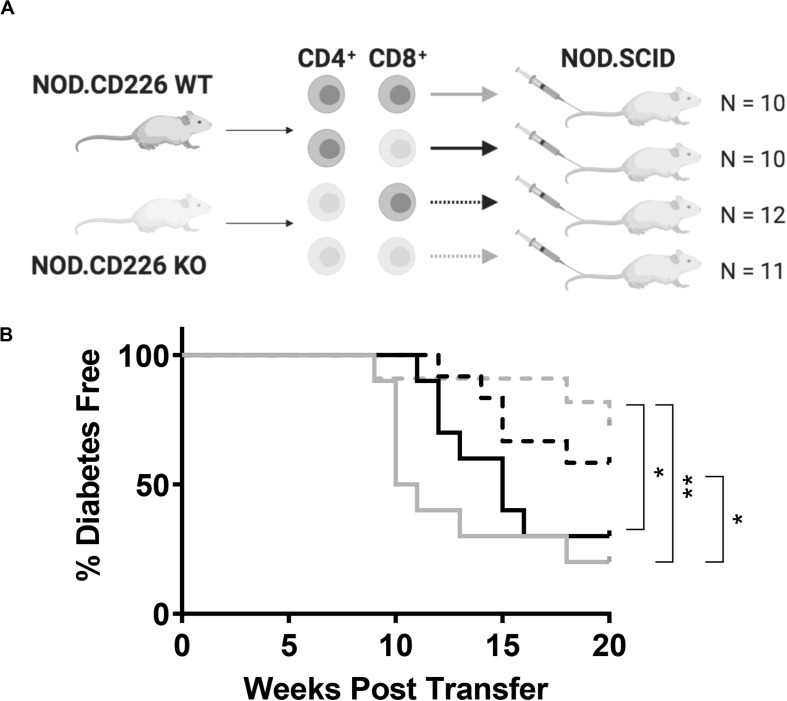
CD226 expression in T cells exacerbates type 1 diabetes induction. **(A)** Schematic of CD4^+^ and CD8^+^ T cell transfer from pre-diabetic CD226 WT or KO mice to female NOD.SCID recipients. Created with BioRender. **(B)** Diabetes induction was monitored for up to 20 weeks post-transfer, with incidence significantly decreased in recipients of KO CD4^+^ and KO CD8^+^ (dashed gray) T cells as compared to WT CD4^+^ and KO CD8^+^ (solid black) or WT CD4^+^ and WT CD8^+^ (solid gray) T cell recipients. Mice receiving KO CD4^+^ and WT CD8^+^ (dashed black) also showed significantly decreased incidence as compared to WT CD4^+^ and WT CD8^+^ T cell recipients. Log-rank (Mantel–Cox) test; *p < 0.05; **p < 0.01. WT CD4^+^ and WT CD8^+^, *n* = 10; WT CD4^+^ and KO CD8^+^, *n* = 10; KO CD4^+^ and WT CD8^+^, *n* = 12; KO CD4^+^ and KO CD8^+^, *n* = 11.

We sought to determine whether the protection from type 1 diabetes seen in the global CD226 KO mouse could be attributed to the altered function of CD4^+^ or CD8^+^ T cells. Here, we transferred combinations of CD226 WT or KO CD4^+^ and CD8^+^ T cells into female NOD.SCID immunodeficient recipients and monitored disease incidence for up to 20 weeks post-transfer. WT CD4^+^ + WT CD8^+^ T cell transfers induced significantly increased disease incidence (*N* = 8/10, solid gray line) as compared to CD226 KO CD4^+^ + CD226 KO CD8^+^ T cell transfers (*N* = 3/11, dashed gray line, *p* = 0.009, Figure 4). WT CD4^+^ + CD226 KO CD8^+^ T cell (*N* = 7/10, solid black line) and CD226 KO CD4^+^ + WT CD8^+^ T cell (*N* = 5/12, dashed black line) transfers showed intermediate disease incidence (Figure 4). However, there was a significant difference in type 1 diabetes development in recipients of CD226 KO CD4^+^ + CD226 KO CD8^+^ T cells versus those in which only CD8^+^ T cells were CD226 KO, suggesting that while CD226 expression on both CD4^+^ and CD8^+^ T cells may contribute toward diabetogenesis in the NOD mouse, the CD226 KO effect is primarily manifest in CD4^+^ T cells in this adoptive transfer setting with a fixed CD4^+^:CD8^+^ T cell ratio.

### CD226 KO Increases Thymic Output of CD8^+^ T Cells in NOD Mice

**FIGURE 5:**
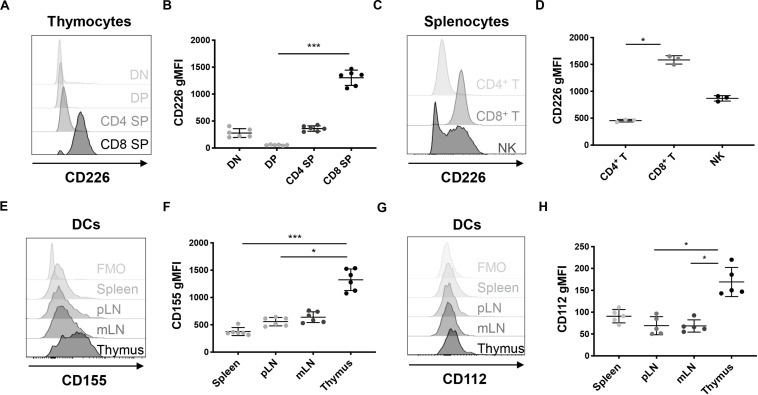
CD226 and ligand expression are enriched on CD8^+^ SP thymocytes and thymic dendritic cells (DCs), respectively. Cells were analyzed from 12-week old pre-diabetic female mice. **(A)** Representative histograms of CD226 geometric mean fluorescence intensity (gMFI) in thymocytes showing that **(B)** CD8 SP thymocytes have higher CD226 expression than DN, DP, and CD4 SP subsets. **(C)** Representative histogram of CD226 expression in spleen. **(D)** gMFI of CD226 elevated on CD8^+^ as compared to CD4^+^ T cells and NK cells. **(E)** Representative histograms of CD155 on CD11c^+^MHCII^+^ DCs from various organs. Fluorescence minus one (FMO) control shown. **(F)** CD155 gMFI is elevated on thymic DCs as compared to DCs from all other organs surveyed. **(G)** Representative histograms of CD112 reveal that **(H)** CD112 expression is highest on thymic DCs. Friedman test with Dunn’s multiple comparisons test; *p < 0.05; **p < 0.01; ***p < 0.001. *N* = +/+, 6 for thymic CD226 and CD155, *N* = +/+, 5 for thymic CD112, *N* = +/+, 3 for splenic CD226 staining.

To elucidate the role of CD226 in central tolerance, we sought to understand how CD226 KO impacted thymocyte development. We assessed CD226 expression in WT NOD thymocyte subpopulations and confirmed that CD8^+^ SP thymocytes showed higher CD226 expression in comparison to CD4^–^CD8^–^ double negative (DN), CD4^+^CD8^+^ DP, and CD4^+^ SP subsets (Figures 5A,B), similar to a previous report (29). CD226 showed elevated expression levels on peripheral CD8^+^ as compared to CD4^+^ T cells in WT female NOD (Figures 5C,D), mirroring CD226 expression in thymocytes (Figures 5A,B) and suggesting preferential CD226 signaling in the CD8^+^ T cell compartment. The ligands of CD226, CD155, and CD112, are reported to be highly expressed on monocyte-derived dendritic cells (DCs) (41), and interestingly, we found that thymic CD11c^+^MHC II^+^ DCs expressed significantly higher levels of both CD155 (Figures 5E,F) and CD112 (Figures 5G,H) as compared to DCs in other secondary lymphoid organs surveyed. These data suggest that the interaction of CD226 on CD8^+^ SP thymocytes with CD155 and CD112 on thymic DCs may play a role in thymic selection.

**FIGURE 6:**
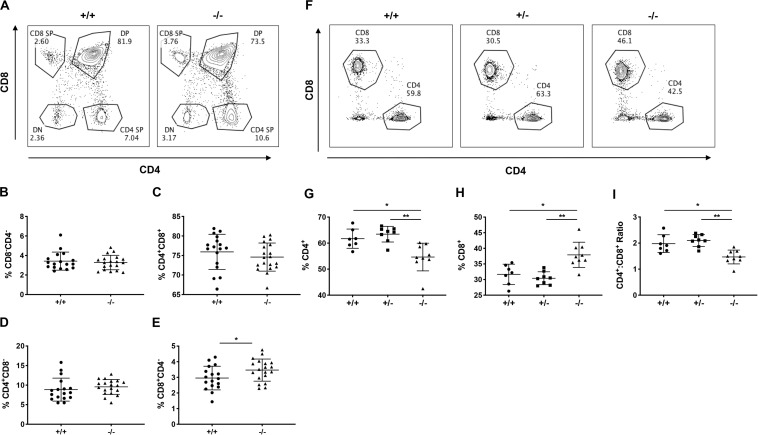
CD226 KO mice have increased thymic output of CD8^+^ T cells. Thymus and spleen were analyzed from 12-week old pre-diabetic female mice. **(A)** Representative flow cytometry plots showing percentage of **(B)** DN, **(C)** DP, and **(D)** CD4 SP thymocytes are similar between WT and KO mice. **(E)** Increased percentage of CD8 SP thymocytes in CD226 KO. Unpaired t test; *p < 0.05. *N* = +/+, 17; –/–, 19. **(F)** Representative flow cytometry plots from spleen, gated on live CD3^+^ T cells. **(G)** Decreased percentage of CD4^+^ and **(H)** increased percentage of CD8^+^ T cells, resulting in **(I)** decreased CD4^+^:CD8^+^ ratio in CD226 KO mice. Kruskal–Wallis with Dunn’s multiple comparisons test; *p < 0.05; **p < 0.01. *N* = +/+, 7; +/–, 8; –/–, 9.

Indeed, 12-week old, pre-diabetic CD226 KO mice showed significantly increased percentages of CD8^+^ SP thymocytes as compared to CD226 WT mice, while DN, DP, and CD4^+^ SP percentages remained similar (Figures 6A–E). We observed a consistent and significant decrease in the percentage of CD4^+^ T cells and increase in the percentage of CD8^+^ T cells in the spleen (Figures 6F–H), leading to a significantly decreased ratio of CD4^+^:CD8^+^ T cells in the spleens of CD226 KO animals (Figure 6I). These data collectively suggest that CD226 signaling may induce the negative selection of CD8^+^ thymocytes, potentially shaping the peripheral CD8^+^ T cell repertoire.

### Decreased Peripheral Activation of CD8^+^ T Cells in CD226 KO Mice

**FIGURE 7:**
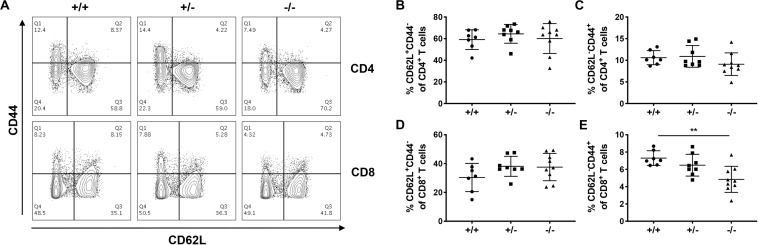
Loss of memory CD8^+^ T cells in the pancreatic-draining lymph nodes of CD226 KO mice. **(A)** Representative flow cytometry plots from 12-week old pre-diabetic females, gated on live CD3^+^ T cells. While percentages of CD62L^+^CD44^–^ **(B)** naïve CD4^+^, CD62L^–^CD44^+^ **(C)** memory CD4^+^ T cells, and **(D)** naïve CD8^+^ T cells remained unaltered in CD226 KO mice, the percentage of **(E)** memory CD8^+^ T cells was reduced in the pancreatic lymph nodes of CD226 KO mice. Kruskal–Wallis with Dunn’s multiple comparisons test; **p < 0.01. *N* = +/+, 7; +/–, 8; –/–, 9.

We next investigated how CD226 impacted peripheral tolerance by analyzing the naïve and memory CD4^+^ and CD8^+^ T cell compartments in various organs of 12-week old pre-diabetic mice. In the pLN, the percentages of naïve CD44^–^CD62L^+^ and memory CD44^+^CD62L^–^ CD4^+^ T cells were not altered in CD226 KO mice (Figures 7A–D). However, CD226 KO mice showed a significantly decreased percentage of memory CD44^+^CD62L^–^CD8^+^ T cells in the pLN as compared to WT mice (Figures 7A,E). The impact of CD226 KO on naïve and memory T cell proportions was not a global phenomenon, as these subsets were not significantly perturbed in the mesenteric lymph nodes (mLN, Supplementary Figure S3) or spleen (Supplementary Figure S4). Therefore, CD226 may augment peripheral CD8^+^ T cell activation within the context of organ-specific autoimmunity.

### Reduced Avidity of IGRP-Reactive CD8^+^ T Cells in CD226 KO Mice

**FIGURE 8:**
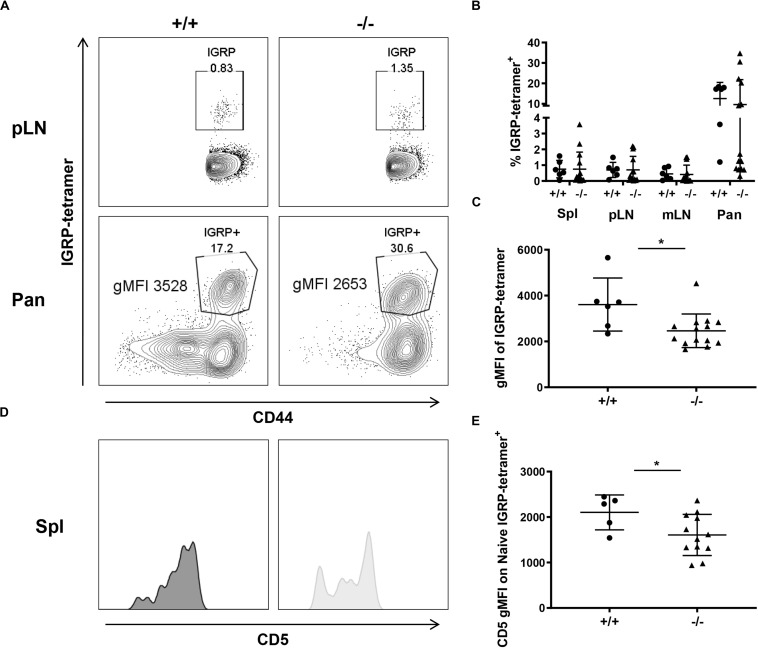
Decreased IGRP avidity in CD226 KO mice. **(A)** Representative flow cytometry plots from 12-week old females, pancreatic-draining lymph node (pLN) gated on live CD8^+^CD44^+^ T cells and pancreas (Pan) gated on live CD8^+^ T cells. **(B)** While percentages of IGRP-tetramer^+^ cells are similar between WT and KO mice for all organs surveyed, **(C)** gMFI of the tetramer within the tetramer^+^ gate is significantly reduced in CD226 KO mice. Mann–Whitney test; *p < 0.05. *N* = +/+, 6; –/–, 12. **(D)** Representative histograms of CD5 expression on naïve IGRP-tetramer^+^ CD8^+^ T cells in the spleen. **(E)** CD5 gMFI on naïve IGRP-reactive cells is significantly decreased in CD226 KO mice. Unpaired *t*-test; *p < 0.05. *N* = +/+, 5; –/–, 12.

To understand the impact of CD226 KO on CD8^+^ T cell autoreactivity, we characterized CD8^+^ T cells recognizing an immunodominant type 1 diabetes epitope (42), islet-specific glucose-6-phosphatase catalytic subunit-related protein (IGRP_206__–__214_) (43). Similar percentages of CD8^+^IGRP-tetramer^+^ T cells were observed within the spleen, pLN, mLN, and pancreas of the CD226 WT and KO mice (Figures 8A,B). However, the geometric mean fluorescence intensity (gMFI) of IGRP-tetramer staining within the IGRP-tetramer^+^ population was significantly decreased in the pancreas of CD226 KO as compared to CD226 WT mice (Figures 8A,C). The magnitude of tetramer binding has previously been shown to correlate with TCR affinity for MHC I-antigen complex in CD8^+^ T cells (44, 45). TCR affinity has also been correlated with CD5 expression levels on mature SP thymocytes and naïve T cells (46). Indeed, CD5 gMFI of naïve IGRP-tetramer^+^ CD8^+^ T cells in the spleen was significantly decreased in the CD226 KO as compared to WT mice (Figures 8D,E). These data suggest that autoreactive CD8^+^ T cells of the CD226 KO mice may possess a decreased functional avidity for IGRP in the context of MHC I.

## Discussion

We show novel evidence that CD226 KO provided protection from type 1 diabetes in NOD mice. Similar proportions of type 1 diabetes seen in CD226 WT and HET mice suggested that a single copy of *Cd226* was sufficient for maximal type 1 diabetes induction. However, TIGIT KO showed minimal impact on type 1 diabetes incidence. A potential explanation for the lack of phenotype observed in the TIGIT KO strain is that there is another inhibitory receptor in the immunoglobulin superfamily, CD96, which can bind to CD155 (47). Thereby, CD96 may compensate for the function of TIGIT in the TIGIT KO animals, allowing for relatively unperturbed T cell function. In support of this notion, combined TIGIT and CD96 blockade has shown increased efficacy at preventing tumor growth via a CD8^+^ T cell-mediated mechanism in a mouse fibrosarcoma model in comparison to either antibody alone (48). Enhancement of CD8^+^ T cell pathogenicity by dual TIGIT and CD96 blockade could therefore be expected to augment type 1 diabetes development in NOD mice.

CD226 KO animals also showed protection from Sjögren’s-associated lacrimal gland inflammation, despite similar extent of salivary gland infiltration. Interestingly, transfer of CD8^+^ T cells alone to NOD.SCID mice can induce lacrimal, but not salivary gland pathology (38). This suggests that the decreased lacrimal disease in the CD226 KO mice may be attributed to CD8^+^ T cell alterations, in agreement with our findings regarding CD226 impacting CD8^+^ T cell numbers, memory formation, and TCR avidity in the context of type 1 diabetes. Whether similar observations of decreased CD8^+^ T cell activation or avidity are seen in the cervical-draining lymph nodes or lacrimal glands of the CD226 KO mice remain unknown.

While we observed that expression of CD226 on both CD4^+^ and CD8^+^ T cells increased the frequency of type 1 diabetes incidence, we did not detect any changes in the thymic development or peripheral activation of CD4^+^ T cells in the absence of CD226. It is likely that CD226 drives pathogenicity in both cell types, and further interrogation of CD4^+^ T cell phenotype in the CD226 KO mouse may uncover mechanisms by which CD226 expression on CD4^+^ T cells contributes to type 1 diabetes. Importantly, the adoptive transfer experiments utilized a 2:1 ratio for CD4^+^:CD8^+^ T cell transfer, while the CD4^+^:CD8^+^ T cell ratio observed in the spleens of CD226 KO mice was closer to 3:2 due to an increase in CD8^+^ T cell output by the thymus. It is plausible that adoptive transfer experiments with this modified ratio may show an enhanced role for CD226 expression on CD8^+^ T cells driving disease, due to sheer increase in the number of autoreactive cells transferred. Regardless, these data suggest that CD4^+^ and CD8^+^ T cell interaction in the global CD226 KO mouse may synergize to drive disease. To further understand the role of CD226 in the immunological mechanisms leading to type 1 diabetes, generation of cell-specific CD226 KO strains are currently underway.

While assessing the impact of CD226 signaling on central tolerance mechanisms, we found increased thymic output of CD8^+^ T cells in the CD226 KO NOD mouse. Others reported that CD226 KO led to decreased proportions of DP and CD4^+^ SP thymocytes in the fetal thymus (29), however, these findings were not replicated in the adult thymus (30), in agreement with our observations in the adult CD226 KO mouse. In contrast to our findings, previous studies have observed that CD226 KO (30) or CD155 KO (31) on the non-autoimmune BALB/c background led to decreased percentages of CD8^+^ SP thymocytes as a possible consequence of early thymic egress, although these studies did not observe differences in peripheral CD8^+^ T cell numbers. These discrepancies may be explained by thymic alterations in NOD as compared to the BALB/c strain (30, 31). NOD mice are known to accumulate thymic medullary giant perivascular spaces (PVSs) with age, and mature CD4^+^ and CD8^+^ SP thymocytes have been found in these compartments (49). The retained SP thymocytes show defects in integrin-type fibronectin receptor expression, thought to impair migration (49–51). Therefore, disruption of CD226 interaction and stalling on Nectin family ligands might preferentially correct CD8^+^ SP thymocyte migration and export to the periphery in NOD mice. Our findings warrant future studies of migration capabilities and numbers of CD8^+^ SP thymocytes in the PVSs of the CD226 KO thymus. Additionally, treatment of NOD mice with CD226 blocking antibody (35, 52, 53) should be utilized to uncouple the effects of CD226 deletion on thymic selection versus peripheral tolerance, while validating our observation of CD226 disruption preventing type 1 diabetes onset.

Our findings suggest that CD226 KO also blunted the peripheral activation of CD8^+^ T cells, particularly in the pLN. These data concur with the previously reported role of CD226 in promoting human CD8^+^ T cell activation, leading to increased cytokine production and cytotoxicity against tumor cells (20). In response to viral infection, CD226 KO mice showed delayed lymphocytic choriomeningitis virus (LCMV) clearance, with reduced IFNγ and TNFα production by virus-specific CD8^+^ T cells (54). Further characterization of the downstream effects of CD8^+^ T cell activation in the CD226 KO NOD model via *ex vivo* analysis of cytokines, perforin, and granzymes (55) or *in vitro* analysis of antigen-specific proliferation and β cell killing (56) are currently underway to further understand the extent to which these mechanisms translate to human type 1 diabetes.

Further supporting the role of CD226 in promoting CD8^+^ T cell pathogenicity in type 1 diabetes, CD226 KO mice showed evidence of reduced TCR avidity of IGRP-reactive CD8^+^ T cells within the pancreas. In agreement with our findings, progression to type 1 diabetes in the NOD mouse has been suggested to rely on avidity maturation of the IGRP-reactive T cell population in the pancreas via preferential expansion of high-avidity TCR clones (57). While costimulatory signaling, including that of CD226 (58), has been implicated in driving selective pressure toward high-avidity T cell clones (59), it is unclear whether our findings in the CD226 KO mouse are reflective of peripheral avidity maturation or, alternatively, a potential consequence of CD8^+^ T cell repertoire modulation at the level of thymic selection. Future work to characterize the thymic output of IGRP-reactive T cells in CD226 KO NOD mice may help to address the mechanisms governing IGRP-specific CD8^+^ T cell avidity. Additionally, the impact of CD226 on CD8^+^ T cell avidity should be validated by measuring tetramer gMFI and CD5 expression on other islet-reactive specificities, such as the AI4-like CD8^+^ T cell pool (60, 61).

We report that CD226 KO attenuates type 1 diabetes via altered thymocyte development in combination with impaired peripheral CD8^+^ T cell activation and IGRP-specific TCR avidity. Our findings here elucidate the mechanisms by which CD226 contributes to type 1 diabetes and support future translational efforts to block CD226 signaling in individuals at-risk for type 1 diabetes and other autoimmune diseases with shared pathogenic mechanisms. Notably, CTLA-4 Ig has been demonstrated to block CD28-mediated costimulatory signaling, with success in recent onset type 1 diabetes at delaying loss of C-peptide (62, 63), endorsing the potential utility of therapeutics such as CD226 blockade in modulating T cell costimulation for the prevention or reversal of type 1 diabetes.

## Data Availability Statement

The raw data supporting the conclusions of this article will be made available by the authors, without undue reservation.

## Ethics Statement

The animal study was reviewed and approved by the University of Florida Institutional Animal Care and Use Committee.

## Author Contributions

MS: writing – original draft, writing – review and editing, formal analysis, visualization, validation, investigation, project administration, and supervision. W-IY: conceptualization, writing – review and editing, formal analysis, visualization, validation, investigation, funding acquisition, project administration, and supervision. JL, JG, CI, and SW: investigation and writing – review and editing. AP and MA: writing – review and editing. MC-T: writing – review and editing, resources, and supervision. SL: writing – review and editing, investigation, formal analysis, validation, funding acquisition, and supervision. DS, AG, and Y-GC: writing – review and editing, resources, and funding acquisition. TB: conceptualization, writing – review and editing, funding acquisition, project administration, and supervision. All authors contributed to the article and approved the submitted version.

## Conflict of Interest

The authors declare that the research was conducted in the absence of any commercial or financial relationships that could be construed as a potential conflict of interest.
