# Supplementary material for: CD226 Deletion Reduces Type 1 Diabetes in the NOD Mouse by Impairing Thymocyte Development and Peripheral T Cell Activation
Source: Front Immunol. 2020 Sep 4;11:2180. doi: 10.3389/fimmu.2020.02180 (PMC7500101; doi:10.3389/fimmu.2020.02180)
Supplement: Supplementary file 1 [file Data_Sheet_1.pdf]

Supplementary Material

1 Supplementary Figures

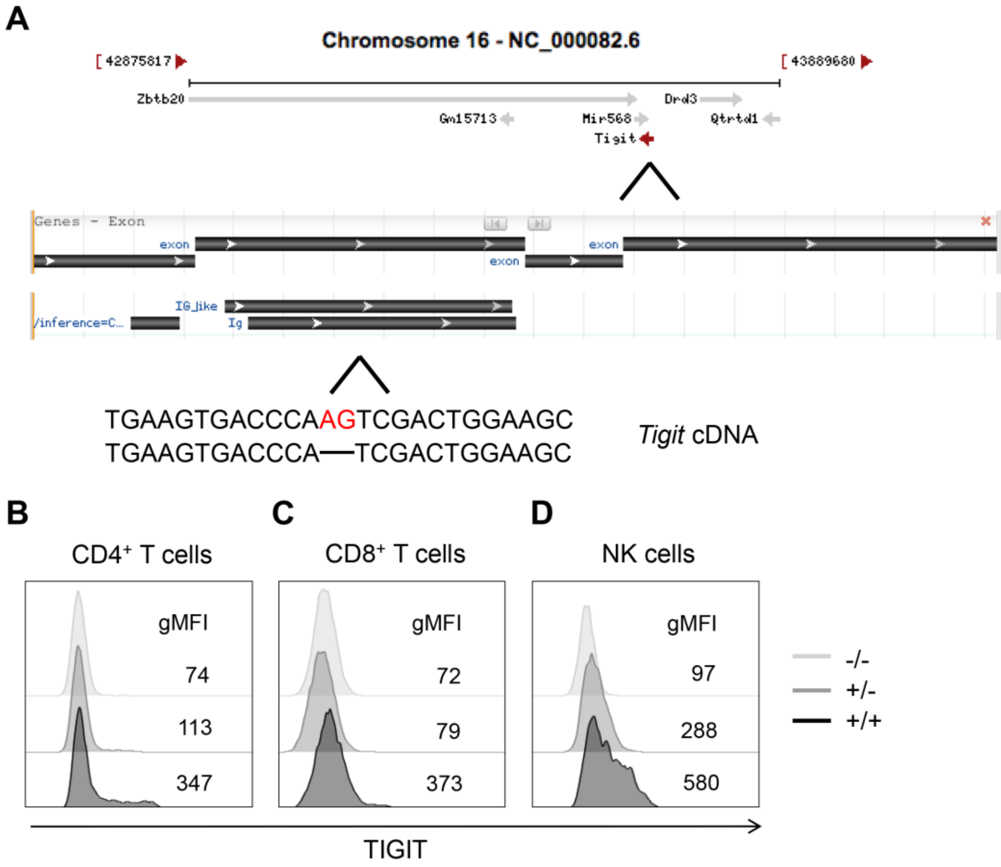

Figure S1. CRISPR/Cas9 targeting methods for generation of TIGIT KO strain. (A) A guide RNA was used to target the second exon of *Tigit*, inducing a two base pair deletion (red) that led to a frameshift and premature termination of TIGIT protein translation. Modified image from Gene NCBI. Histograms showing TIGIT expression in splenic **(B)** CD4^{+} T cells, **(C)** CD8^{+} T cells, and **(D)** NK cells of 10-week-old female, prediabetic WT (black), HET (gray), and KO (light gray) mice.

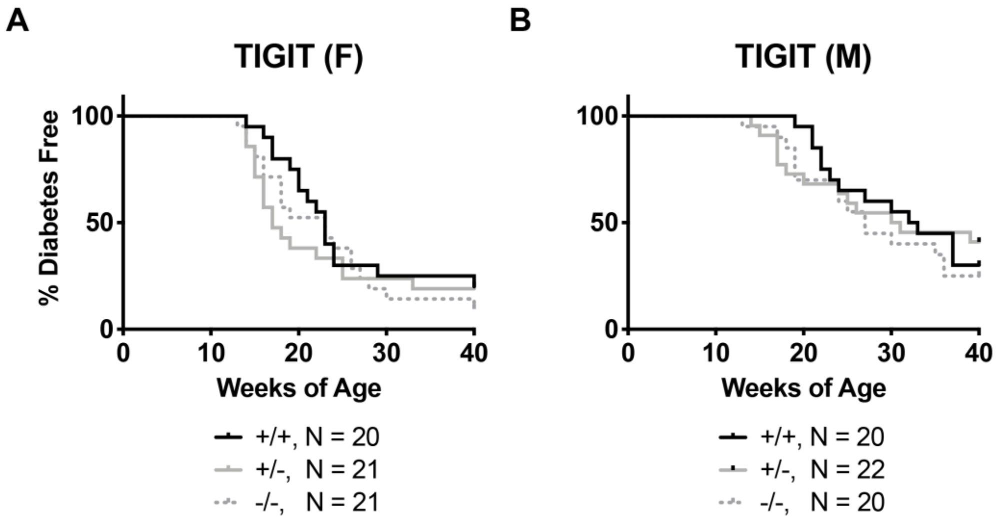

Figure S2. TIGIT KO does not modulate type 1 diabetes incidence in NOD mice. Disease incidence was monitored weekly, with diabetes defined as two consecutive daily blood glucose readings > 250 mg/dL. TIGIT KO (dotted lines) shows similar disease incidence in **(A)** females and **(B)** males as compared to WT (solid black) or HET (solid gray) mice. Female: +/+, n = 20; +/-, n = 21; -/-, n = 21; Male: +/+, n = 20; +/-, n = 22; -/-, n = 20.

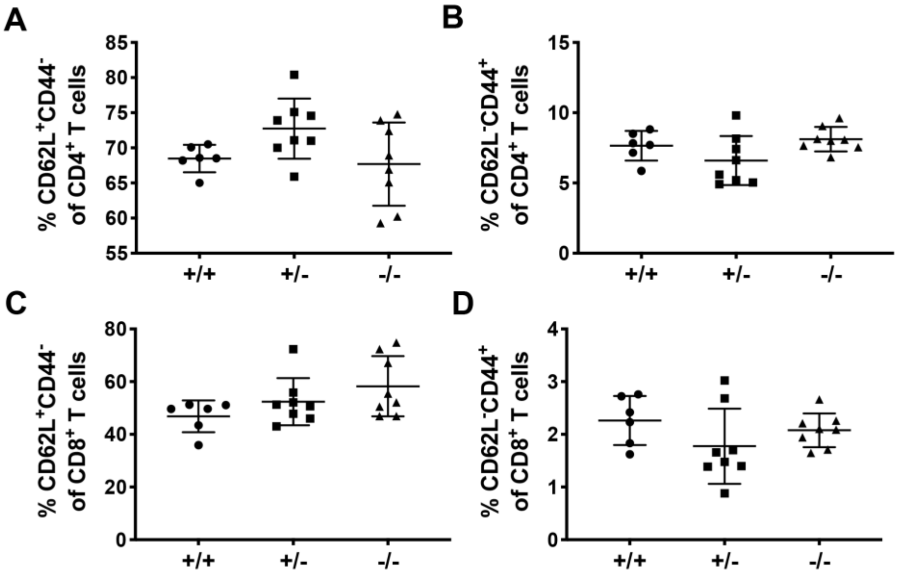

Figure S3. Naïve and memory T cell percentages were unchanged in the mesenteric lymph nodes of CD226 KO mice. Data from 12-week old pre-diabetic females. Percentages of **(A)** naïve $CD4^+$, **(B)** memory $CD4^+$, **(C)** naïve $CD8^+$, and **(D)** memory $CD8^+$ T cells unchanged in the mesenteric lymph nodes of CD226 KO mice. N = +/+, 7; +/-, 8; -/-, 9.

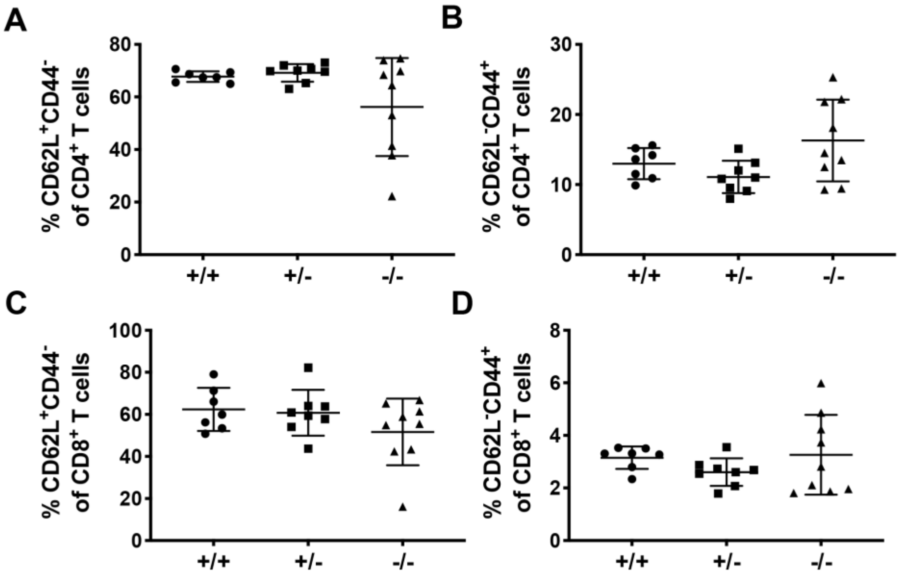

Figure S4. Naïve and memory T cell percentages not significantly different in the spleen of CD226 KO mice. Data from 12-week old pre-diabetic females. Percentages of **(A)** naïve $CD4^+$, **(B)** memory $CD4^+$, **(C)** naïve $CD8^+$, and **(D)** memory $CD8^+$ T cells unchanged in the spleens of CD226 KO mice. N = +/+, 7; +/-, 8; -/-, 9.
